# Color by Soy: Genistein Linked to Epigenetic Effects

**Published:** 2006-04

**Authors:** Bob Weinhold

There is substantial evidence that a pregnant mother’s exposure to environmental substances can affect her young. Now researchers have uncovered the first direct evidence that maternal exposure to a phytoestrogen in soy can cause lifelong epigenetic changes—that is, changes in gene activity from processes other than changes in the DNA sequence—in mouse offspring **[*EHP* 114:567–572; Dolinoy et al.]**. If the findings hold up, there eventually could be repercussions in several important arenas, including recommendations about what pregnant women and infants should eat.

The researchers made their observations in genetically identical yellow mice. The mothers in the test group ate a diet modified to include a concentration of genistein typical of what people eating a high-soy diet would consume, while the control group mothers ate the same food without the genistein. The diets began two weeks before mating and continued through pregnancy and lactation. At 21 days after birth, the offspring were weaned to a stock maintenance diet, which they ate for the rest of the study period.

The researchers assessed offspring coat color and body weight, traits that can vary with methylation (a mechanism in which methyl groups attach to DNA where cytosine bases occur consecutively). The offspring exhibited wide color differences, ranging from yellow to brown, with varying degrees of mottling in between. The researchers found that these differences corresponded closely with methylation in a DNA region upstream of the offspring’s *Agouti* gene, which determines coat color. The 44 genistein-fed offspring were more than twice as likely as the 52 controls to have brown fur and to have much higher methylation, while they were only one-third as likely to have yellow fur and much lower methylation. Animals that had a blend of yellow and brown fur had progressively increased methylation as brown became more dominant.

For weight gain, the brown mice showed by far the least propensity to become overweight. The other groups, with yellow or various combinations of yellow-brown fur, had roughly the same trend toward becoming significantly overweight. The researchers also found that methylation was evident in many parts of the body, including the brain, liver, kidney, and tail.

The team speculates that the ability of genistein to increase DNA methylation provides a plausible explanation for the lower incidence of certain cancers in Asians compared to Westerners. Nevertheless, the pathway by which these changes occurred suggests there could also be significant adverse interactions between genistein and common dietary supplements such as folic acid, which is added to many foods and recommended for pregnant women. Soy-based infant formulas might also be a concern because of the high levels of genistein present in these products. More research is needed to determine if these worrisome possibilities are correct.

## Figures and Tables

**Figure:**
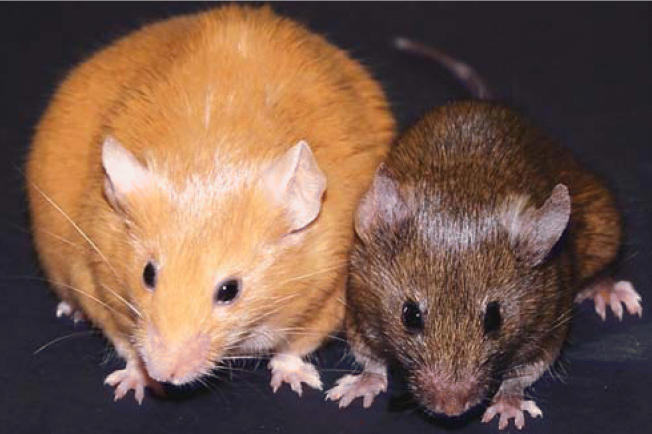
Telltale traits. Differences in the size and color of offspring are epigenetic effects of genistein consumption (via a high-soy diet) by mouse moms.

